# Fostering green transformational leadership: the influence of green educational intervention on nurse managers’ green behavior and creativity

**DOI:** 10.1186/s12912-024-01991-0

**Published:** 2024-06-07

**Authors:** Manal Saleh Moustafa Saleh, Hanan Elsaid Elsabahy, Sahar Abdel-Latif Abdel-Sattar, Zaineb Naiem Abd-Elhamid, Abdulellah Al Thobaity, Sahar Mohammed Mohammed Aly, Wafaa Mohamed Shokry

**Affiliations:** 1 Nursing Administration, Faculty of Nursing, Zagazig University, Zagazig, Egypt; 2 Nursing Department, College of Applied Medical Science, Shaqra University, Shaqra, Saudi Arabia; 3 Nursing Administration, Faculty of Nursing, Mansoura University, Mansoura, Egypt; 4 Medical Surgical Department, Taif University, Taif, Saudi Arabia; 5 Nursing Administration, Faculty of Nursing, Port Said University, Port Said, Egypt; 6 Nursing Administration, Faculty of Nursing, Menoufia University, Menoufia, Egypt

**Keywords:** Green transformational leadership, Green behavior, Green creativity, Nurse managers

## Abstract

**Aim:**

This study aimed to investigate the influence of green transformational leadership educational intervention on nurse managers’ green behavior and creativity.

**Background:**

Organizational creativity is greatly influenced by leaders and their personality attributes. Additionally, innovative employee behavior is crucial for organizational performance and survival, which in turn promotes long-term organizational growth.

**Method:**

A quasi-experimental design was conducted by using pre-test, post-test, and follow-up for a group that included 116 nurse managers who completed the intervention. Data were collected through the green transformational leadership knowledge questionnaire, green transformational leadership scale, green behavior questionnaire, and green creativity scale.

**Results:**

Following the implementation of the Green Transformational Leadership educational intervention, there was an improvement in responses connected to the nurse manager’s use of green behavior and creativity. Three months after the intervention ended, the improvement was still present.

**Conclusion:**

Nurse managers who had good knowledge about green transformational leadership showed increased green behavior and green creativity, which enhanced the organization’s success. This study showed the significance of developing and improving the skills of managerial creativity for the nurse supervisor of a hospital through training in transformational leadership.

**Implications for nursing management:**

The concept of “green transformational leadership” refers to leadership behaviors and strategies aimed at promoting environmental sustainability and responsibility within an organization or a specific context. In the case we mentioned, it involves implementing educational interventions targeted at nurse managers to enhance their understanding and adoption of green practices, as well as fostering green behavior and creativity among them.

**Supplementary Information:**

The online version contains supplementary material available at 10.1186/s12912-024-01991-0.

## Introduction

Green practices have been integrated into various organizational aspects from green leadership to green product and/or process practices. Organizational researchers have recently focused on the green behavior of employees since data suggests that individual employee conduct can have a significant impact on and improve the organization’s environmental performance. Green employee behavior refers to employee actions that improve or worsen environmental sustainability, whether they are voluntary or involuntary and measurable [1]. Green transformational leadership is described as “behaviors of nurse managers that encourage subordinates to perform above expected levels of environmental performance and inspire subordinates to attain environmental goals. It encourages nurse managers to be aware of environmental issues” [2, 3].

Idealized influence, inspirational motivation, intellectual stimulation, and individualized consideration were employed as the four qualities of green transformational leadership. The leadership connects with clients and rewards them for green behavior, which is crucial for inspiring employee motivation [4, 5]. Green transformational leadership, theory-based evidence, and experience-based evidence show that personal and environmental context are some of the aspects that can influence employees’ tendency towards environmentally friendly conduct at work. In this context, researchers identify transformational leadership as a key element in the development of environmentally friendly workplace behavior [6].

Green transformational leadership directly affects leaders’ green behaviors. Green transformational leaders influence employees through their green plans, visions, goals, beliefs, and ideas. The green behavior concept emerged from the environmental issues in the 1960s [7]. Green transformational leadership help leaders understand the importance and feasibility of green behaviors to realize that green behaviors are encouraged and expected by organizations and that they should demonstrate green behaviors. Green behaviors include reducing waste and recycling in their organization. They can contribute positively to climate change by feeling sensitive towards cleaning the harmful gases that are released into the environment or polluted waters [8]. Green behavior has been defined as “scalable actions and behaviors that employees engage in that are linked with and contribute to or detract from environmental sustainability” [9]. Therefore, scholars often explain employee green behavior as employee pro-environmental behavior, employee work-green behavior, environmental green behavior at the workplace, and employee green behavior [10]. Green behavior has referred to all beneficial workplace activities aimed to assist the environment, such as energy and water conservation [11].

Any action that benefits the environment or lessens environmental damage is referred to as “green behavior.” The green behavior of employees in the workplace has received more attention as an enterprise sustainable development strategy [12]. Green conduct is viewed by nursing managers as a personal trait that is aligned with the objectives of environmentally sustainable growth, and it is promoted by leaders who exhibit positive green behavior [13]. Future studies on nurse managers’ green conduct are encouraged by the researchers, as it is defined as an aspect of environmentally friendly behavior within organizational transformational leader behavior and is thought to be a crucial element of organizational sustainable development [14].

Green behavior among nurses can immediately decrease expenses while also protecting the natural environment and organizational sustainability by conserving materials and energy [11]. There is a framework for measuring green behavior across three dimensions: green learning, individual practice, and influence over others. We discovered that there is a gap between the ability and awareness of employees when implementing green behavior. It can be difficult for people to implement green behavior properly. Therefore, enhancing individual green learning is particularly crucial. Additionally, a majority of workers think that green learning can both improve their own capacity for green conduct and contribute to greater public awareness of the adoption of green behavior [15].

Organizational innovation is significantly influenced by leaders and their characteristics [16]. By encouraging, supporting, lending a helping hand, and boosting their confidence to take on new ideas, transformational leaders foster employee creativity [17]. As a result of staff’ creativity, a firm may learn new things that could help it develop, succeed, and have a better chance of surviving. According to [18], the outcomes of novel ideas and enhanced tasks take the form of new products and improved work processes in the instance of nurse leaders [19].

Several research have supported and provided evidence to support the claim that green transformative leadership significantly impacts creativity. According to a thorough review of the literature, there are four dimensions to transformational leadership: intellectual stimulation, individualized consideration, charisma, and inspirational motivation. Because of their capacity for intellectual stimulation, transformational leaders are able to stimulate the cognitive capacities of their employees, which in turn fosters the growth and development of those employees’ creative skills [20–22].

Green creativity places a stronger emphasis on the sustainability and environmental friendliness of goods, services, and behaviors. Employee creativity plays a crucial role in organizations as well. Nursing manager creativity is the manager’s originality or fresh ideas. To make this happen, transformational leadership must have an impact. Transformational leadership demonstrates that it can promote leader creativity by encouraging it in the workplace [23, 24].

### Significance of the study

The need to use natural resources more effectively by fostering sustainable development has arisen as a result of the fast growth, industrialization, and economic development occurring around the world [25]. Numerous research studies have emphasized the promotion of green creativity methods in manufacturing as a means of achieving long-term environmental goals. The essential strategy for encouraging creativity among followers is leadership. To this purpose, transformational leadership entails management that encourages workers’ inventiveness through energizing and group-goal-oriented conduct. By taking a creative approach to resolving environmental problems and encouraging an atmosphere where people may express their fullest level of creativity, green transformational leadership motivates staff to meet corporate goals [26].

Previous research has shown that green transformational leadership positively affects organizational greenness, which encourages green creativity inside the organization. Therefore, in order to combat the mounting threat of climate change, it is necessary to encourage employees to engage in eco-friendly practices at work [9, 27]. By creating an environment that is favorable and supportive of organizational behavior that is green, transformational leadership plays a significant role [28]. Additionally, it is asserted that transformational leadership is a major factor in encouraging staff innovation [27].

Followers develop inspiring ideas as a result of the transformational leader’s charm, earning them respect and subsequently their allegiance. Instilling a sense of connection in their followers through personalized consideration enables the transformational leader to foster the growth of mutual care [28].

In addition, the transformational leader describes the path through which the vision can be realized with the help of inspirational motivation and giving the organization a clear direction. The ability of a transformational leader to stimulate the mind enables them to inspire the cognitive talents of their followers, which fosters the expansion and development of followers’ creative capacities. Additionally, transformational leaders foster employee creativity by empowering them to take on new initiatives and encouraging, assisting, and supporting them in doing so [26, 29].

### Aim of the study

#### Primary aim

- To investigate the influence of green transformational leadership educational intervention on nurse managers’ green behavior and creativity.

#### Secondary aim

- To evaluate nurse managers’ knowledge of green transformational leadership skills.
- To investigate nurse managers’ behavior toward green transformational leadership before and after educational intervention.
- To evaluate the effectiveness of green transformational leadership educational intervention on green behavior and green creativity among nurse managers.

**Hypotheses of the study**:

1. Nurse managers’ knowledge about green transformational leadership will have improved after educational intervention.
2. Nurse managers’ behavior toward green transformational leadership will have improved after educational intervention.
3. Green transformational leadership educational intervention positively relates to green behavior among nurse managers after intervention.
4. Green transformational leadership educational intervention positively relates to green creativity among nurse managers after intervention.

### Theoretical framework

#### Green transformational leadership and nurse managers’ green behaviors

Green transformational leadership had an impact on the green behaviors of the nurse manager; but, because leaders set an example for others, transformational leaders can also have an impact on the green behavior of those they follow by modeling green behavior. Additionally, managers who serve as role models for environmental issues and inspire their staff to follow suit by setting an example for green behavior are exhibiting the green idealized effect [30]. Like task-oriented leaders, green transformational leaders are focused on long-term, sustainable growth through encouraging people to act in a more environmentally friendly manner and integrating their own green values with the organization’s green values. This showed that transformational leaders have a significant role in driving employees’ behavior via inspiration, motivation, and satisfaction. The green transformational leaders inspired and promoted environmental behaviors, making it possible for staff members to understand the value of green behavior and that the company expects and supports it [31].

#### Green transformational leadership and nurse managers’ green creativity

The creative thinking of employees is positively impacted by transformational leadership when it comes to promoting green production. Green transformative leaders encourage creative thinking by motivating subordinates to express their opinions and gain a passion for ideas [32]. Also, present green opportunities and corporations can gain a competitive edge by projecting a more environmentally conscious image. Fostering staff members’ green creativity to generate innovation is one of nurse managers’ top priorities. In addition to providing the necessary context-specific tools to allow staff members to express their creative expectations, green transformational leadership cultivates a positive, supportive environment [33, 34]. Managers’ green creativity is enhanced by green transformational leadership which supporting, encouraging, extending a helping hand, and building their confidence to take new initiatives. Green creativity, as an important component of workplace creativity, may be considered as the same activity. If an organization aspires to be more environmentally friendly while simultaneously empowering staff to participate in activities like training, it must create a structure that gives workers, such as trainees and opportunities [35, 36].

## Method

### Design, setting, and subject of the study

A quasi-experimental design was conducted using pre-test, post-test, and follow-up for one group. The study was conducted at Main Mansoura University Hospital (MMUH) and its three medical centers: the convalescence center, the specialized medical center, and the center of plastic and burn surgery.

All available nurse managers who were on duty at their place of work at the time of data collection were included in the study by using a convenience sample technique.

### Sample size

Based on a review of past literature [37], reported that there was a high statistically significant negative correlation between challenges faced by nursing managers and their perception of organizational support (*p* < 0.001). The sample was calculated at power 80% and confidence level 95%, with the following equation N = [ Zα + Zβ / C] 2 + 3 where: Zα = The standard normal deviate for α = 1.9600. The Zβ = standard normal deviate for β = 0.8416 C = 0.5 * ln[(1 + r)/(1-r)] = 0.1789. The calculated sample was 105 and it was increased by 10% to avoid dropout so the total sample was 116 participants. All available nurses were first-line managers, or supervisors, who were on duty at their place of work at the time of data collection. They were included in the study if they agreed to participate in the research. Accordingly, 86 first-line nurse managers and 30 nurse supervisors agreed to participate in this research.

### Inclusion criteria

Nurse managers of different ages, genders, and years of education, who had at least two years of experience, were invited to participate in the research. However, male nurse managers were few in the Mansoura University Hospital because the male gender have joined the nursing profession recently, thus few of them hold managerial positions.

### Exclusion criteria

If the experience of nurse managers is less than two years.

### Data collection

#### Instrument one

The Green Transformational Leadership Knowledge Questionnaire (GTLKQ) was developed by the investigator. This instrument was used to evaluate nurse managers’ knowledge about green transformational leadership before and after educational intervention. (S F -1)

The GTLKQ was included in Personal data (6 items) including age, qualification levels, experience, marital status, work unit, and whether or not they have heard about green leadership and Transformational Leadership Knowledge Questionnaire which consisted of 10 multiple choice questions.

#### Green transformational Leadership Knowledge Questionnaire scoring system

The ten transformation leadership knowledge items were measured with a three-point Likert scale developed by the investigator as (0–2) with (0) for an Incorrect answer, (1) for a Correct but incomplete answer, and (2) for a Correct and complete answer. Summing these answers has yielded a total score for the GTLKQ in the range of 0–20. Accordingly, if the total knowledge score was 0–11, he/she was classified as having Poor Kn. If the total score was 12–15, he/she was classified as having Moderate Kn. And if the total score was 16–20, he/she was classified as having Good Kn. (S F -1)

**Instrument two**: [38] developed the Green Transformational Leadership Scale, which the investigator adapted to assess nurse managers’ perceptions toward green transformational leadership before and after educational intervention. It comprised six items and was scored on a five-point Likert scale, with 1 representing strongly disagree and 5 representing strongly agree.

#### Green transformational Leadership Scale scoring system

Six items were scored with a five-point Likert scale (1–5) with (1) for Strongly disagree, (2) for Disagree, (3) for Neutral, (4) for Agree, and (5) for Strongly agree. Adding these individual scores yielded a score for the questionnaire in the range of 6–30.

**Instrument three**: The Green Behavior Questionnaire (GBQ) was created by Zhang, Yang, Cheng, and Chen [38], and was adopted by the research team to assess nurse managers’ green behavior before and after implementing an educational intervention. The adapted GBQ was made up of 13 items divided into four dimensions: (a) green learning (3 items), (b) individual practice (4 items), (c) influencing others (3 items), and (d) organizational voices (3 items). Each item was scored using a five-point Likert scale, with 1 representing strongly disagree and 5 representing strongly agree.

#### Green Behavior Questionnaire scoring system

13 questions were scored on a five-point Likert scale (1–5) with (1) representing strongly disagree, (2) representing disagree, (3) representing neutral, (4) representing agree, and (5) representing strongly agree. These results were added yielding a questionnaire being graded on a scale of 13 to 65.

#### Instrument four

The researcher modified [39, 40] Green Creativity Scale (GC) to assess nurse managers’ green creativity both before and after the implementation of an educational intervention.

#### Green Creativity Scale scoring system

Six items were captured using a five-point Likert scale (1–5) with (1) for Strongly disagree, (2) for Disagree, (3) for Neutral, (4) for Agree, and (5) for Strongly agree. The questionnaire was evaluated giving a score of 6–30.

### Translation procedures and the tool’s validity and reliability

The four instruments of green transformational leadership knowledge, green transformational leadership, green behavior, and green creativity scale were translated into Arabic using the process of translation and back-translation [41]. The translated tools were reviewed by a panel of seven professors/assistant professors from the university’s nursing academic staff. Furthermore, the panel examined the measurement tools for back translation from Arabic to English and content and face validities. Nothing required modifications based on the panel’s recommendations. Utilizing reliability analysis, the instrument’s internal consistency was evaluated.

Reliability was estimated among 10 manager nurses using the test-retest method two weeks apart. Then Cronbach alpha reliability test was done using the SPSS software. Cronbach’s alpha coefficients were calculated for multipoint items to evaluate the measurement reliability. Cronbach’s alpha for each instrument was as follows: 0.84 for the first instrument *(Green Transformational Leadership Knowledge Questionnaire)*, 0.93 for the second instrument *(Green Transformational Leadership Scale)*, 0.86 for the third instrument *(Green Behavior Questionnaire*), and 0.90 for the fourth instrument *(Green Creativity Scale).* The results of Cronbach alpha reliability test results for the four instruments indicated that they were reliable in detecting the objectives of the study.

### Pilot study

Furthermore, an initial pilot study was conducted with 16 (13%) participants of the study population. The pilot study evaluated the study instruments’ readability, applicability, and time demand as well as their viability. The study results remained unchanged after the pilot study’s findings were integrated.

### Procedures

The researchers started collecting data from the nurse managers who met the inclusion criteria after receiving legal authorization (Ethical and Research Committee Decision No. 881–2022 and Hospital Administration approval,10- 2022). Researchers have met participants and held Telegram groups to obtain their cooperation, and verbal consent to be included in the study. Also, for ease of communication, appropriate session times were set and posters and videos related to the program sent. The study was carried out through interviewing, implementation, and evaluation.

#### Interviewing

Interviewing started at the end of August 2022 and ended at the end of September 2022. The educational program, its goal, the intervention, and the period of intervention one-month and three-month requirements were all explained to the study participants. The educational intervention was run twice. The total number of nurse managers was 116. So, it was divided into two groups based on the departments they worked in each time, each group consisted of between 50 and 60 nurse managers. The total duration (12 h theory) for each group was divided into six sessions with two hours for each session. The educational intervention was implemented in six sessions for each group and three sessions were conducted weekly. The intervention lasted for two weeks for each group (one month for two groups). Various methods of teaching (lecture, discussion, role-playing, brainstorming, video material, etc.) were utilized.

#### Implementation

Data was gathered by meeting nurse managers and explaining the study’s goal to them. They received assurances that the data would only be utilized for the study and that it would be used for scientific studies. To gather information about the nurse managers’ level of knowledge regarding green transformational leadership, a knowledge questionnaire was given to all of the nurse managers before the educational intervention started, after it had finished, and three months later.

Prior to creating the sessions, the researchers looked over a large body of research and evidence-based literature. Green transformational leadership, green behavior, and green creativity were all the subjects of interventions that were produced after a review of the literature, an analysis of assessment findings, and an evaluation of increasing gaps. (Details about the program S.F.2)

#### Evaluation of green transformational leadership educational intervention phase

At the conclusion of the educational intervention and three months later, nurse managers were given a chance to reassess their understanding of green transformational leadership and compare their results with the pre-test. The purpose, mediator, and three-month program results of the green transformational leadership educational intervention on the environmentally conscious actions and green thinking of nurse managers were evaluated. A time in December 2022 was used to fill in the follow-up questionnaire.

### Statistical approach

Data was altered and coded to fit into a form that was specifically made for computer entry. The SPSS (Statistics Package for Social Science) package, version 22, was used to enter and analyze the data. Excel was used to create the graphics.

Quantitative data were presented by mean (X) and standard deviation (SD). A comparison of mean practice scores was made among nursing directors and supervisors’ pre-intervention and immediate post-intervention. Also, a comparison of mean practice scores among nursing directors and supervisors’ pre-intervention and follow-up was done using paired t-tests. In addition, a comparison was made of mean practice scores among head nurses’ pre-intervention and immediate post-intervention. Also, a comparison of mean practice scores among head nurses’ pre-intervention and follow-up was done by using a paired t-test. Similar patterns were used in green behavior by using paired t-tests as well as green creativity using paired t-tests.

Qualitative data related to knowledge about green transformational leadership skills, quantity, and percentage were displayed as frequency distribution tables. Chi-square analysis was used to examine it. The Fisher Exact test (if the table had four cells) or the Likelihood Ratio (LR) test (if the table had more than four cells) was applied, if the anticipated value of any cell in the table was less than 5. For all significant tests, the level of significance was fixed at *P* < 0.05.

## Results

Table 1 Contains personal characteristics of studied nurse managers and shows that the nurse managers age was 40.2 ± 5.7 Y. All of the participants were female (100%) and the majority (96.6%) were married. Most nurse managers had a bachelor’s degree (90.5%) and the majority were first line managers (74.1%), while the lowest (25.9%) were nursing supervisors.

Table 2 Demonstrates the effect of the instructional intervention program on nursing managers’ percentages of green transformational leadership knowledge when measured before the educational intervention, immediately post-intervention, and follow-up three months later. Both immediate post-intervention and follow-up results revealed a highly significant improvement in the green transformational leadership knowledge levels (*p* < 0. 001). The immediate post-intervention ‘good knowledge’ responses increased from 2.6% pre-intervention to 93.1% immediate post-intervention. The follow-up intervention program ‘good knowledge’ responses increased from 2.6% pre-intervention to 90.5% at follow-up.

Table 3 provides a comparison of green transformational leadership knowledge among nurse managers at pre-intervention, post-intervention, and follow-up. This table shows that the overall knowledge score of nurse managers increased significantly at post-intervention and follow-up (19.7 and 19.3; respectively) compared to 2.1 in the pre-intervention. In addition, there was a highly significant difference between post and follow-up intervention for each of the nurse supervisors and head nurses.

Table 4 demonstrates the efficacy of the green transformational leadership educational intervention for the green transformational leadership practice of nurse managers. Post-intervention and follow scores revealed a highly significant improvement (*p* < 0. 001) in green transformational leadership practice. Among nursing supervisors, the immediate post-intervention total mean practice increased from 14.5 ± 1.8 pre-intervention to 28.3 ± 1.02 post-intervention, and the difference was highly significant statistically (*P* < 0. 001). A similar trend was observed among head nurses, where the immediate post-intervention total mean practice increased from 13.1 ± 1.1 pre-intervention to 28.6 ± 0.9 post-intervention, and the difference was highly significant statistically (*P* < 0. 001). Similar trends were observed among nurse managers concerning the pre-green transformational leadership intervention and follow-up mean practices, and the difference was highly statistically significant (*p* < 0. 001) for nursing supervisors as well as head nurses.

Table 5 presents mean scores of the green behavior dimensions and total green behavior, as well as green creativity among nurse managers at pre-intervention, immediate post-intervention, and follow-up. This table shows a statistically significant difference between nurse managers’ knowledge of green behavior’ dimensions and total green behavior between pre-and post-intervention and between pre-intervention and follow-up. Nurse manager’s knowledge levels increased after the intervention. Moreover, there was a large effect of the intervention on nurse managers’ knowledge. Also, there was a statistically significant difference between nurse managers’ knowledge between post-intervention and follow-up.

**Fig. 1:**
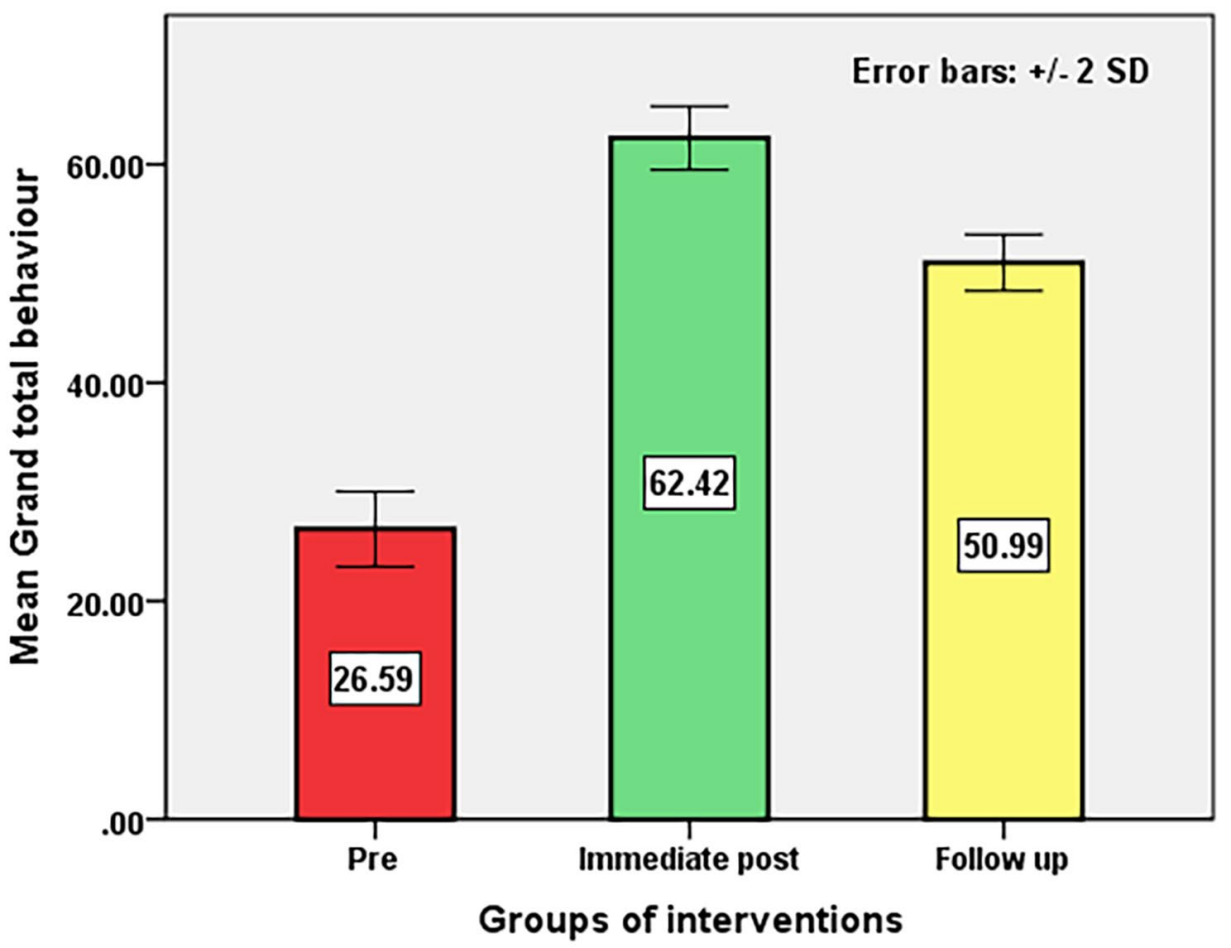
Effect of Green Transformational Leadership educational intervention program on nurse managers’ green behavior before, immediately post and follow up intervention (*N* = 116)

Table 6; Fig. 1 show the level of significance and total mean score regarding pre-intervention, post-intervention, and follow-up green transformational leadership on green behavior among nurse managers. This highlighted the efficacy of the green transformational leadership educational intervention on the green behavior of the nurse managers. Post-intervention and follow-up revealed a highly significant improvement (*p* < 0. 001) in the different dimensions of green behavior as well as total green behavior. The mean difference between pre- and immediate post-intervention total behavior responses = -35.8, with SD = 2.2, and 95%. Confidence interval of the difference − 36.2 to − 35.4. A similar trend was observed concerning the pre-intervention and follow-up on green behavior responses, and the difference was highly statistically significant (*p* < 0. 001) for each. This result confirmed this study’s third hypothesis that “green transformational leadership educational intervention positively relates to green behavior among nurse managers after intervention”.

**Fig. 2:**
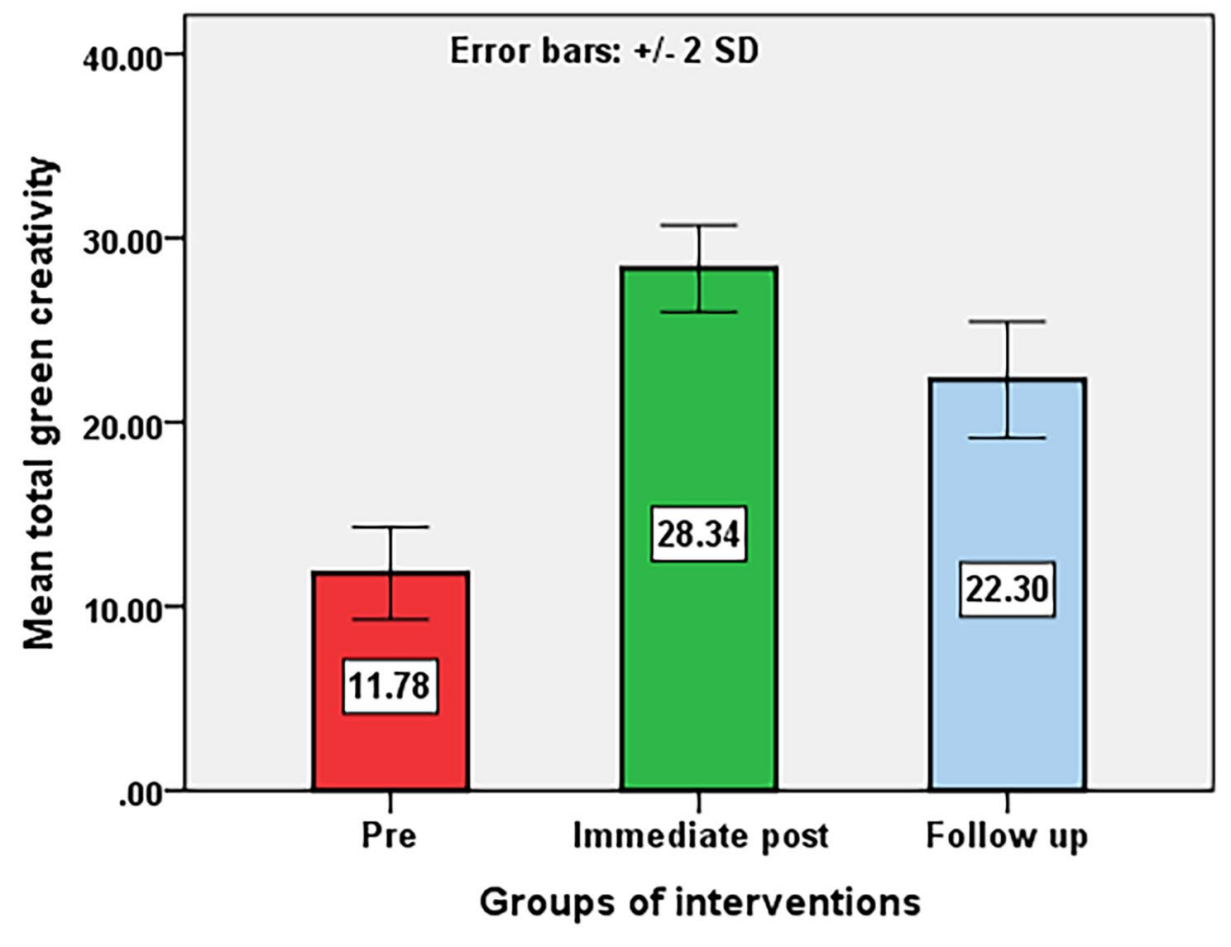
Effect of Green Transformational Leadership educational intervention program on nurse managers’ green creativity before, immediately post, and follow-up intervention (*N* = 116)

Table 7; Fig. 2 show the level of significance and total mean score regarding pre-intervention, post-intervention, and follow-up green transformational leadership on green creativity among nurse managers. This highlighted the efficacy of the green transformational leadership educational intervention on the green creativity of the nurse managers. Post-intervention and follow-up scores revealed a highly significant improvement (*p* < 0. 001) in the different dimensions of green creativity as well as for total green creativity. The mean difference between pre and immediate post-program total behavior responses = -35.8, with SD =(2.2), and a 95%. Confidence interval of the difference = − 36.2 to − 35.4. A similar trend was observed concerning the pre-intervention and follow-up scores for green creativity, and the difference was highly statistically significant (*p* < 0. 001) for each. This result confirmed this study’s fourth hypothesis which stated “green transformational leadership educational intervention positively relates to green creativity among nurse managers after intervention”.

**Table 1:** Personal characteristics of studied nurse managers

Personal data	*N* (%)	
	N0.	%
Age (Mean ± SD)	40.2 ± 5.7 Y	
**Marital status**:		
Married Unmarried	112 4	96.6 3.4
**Qualification**:		
Master Bachelor	11 105	9.5 90.5
**Nursing managers**:		
First-line managers Nurse Supervisors	86 30	74.1 25.9
Experience (Mean ± SD)	16.1 ± 5.2 Y	
**Heard about green leadership**:		
No Yes	95 21	81.9 18.1

**Table 2:** The efficacy of the instructional guideline’s intervention program on the knowledge of the nursing managers about green transformational leadership levels as well as total mean knowledge scores (*N* = 116)

Green leadership knowledge levels	Pre-Intervention (*N* = 116)		Immediate post-intervention (*N* = 116)		Follow-up Intervention (*N* = 116)		*X*^2^*P* value
	*N*	%	*N*	%	*N*	%	
Poor knowledge	102	87.9	0	0	1	0.9	= 299.8, *P* < 0. 001
Moderate knowledge	11	9.5	8	6.9	10	8.6	
Good knowledge	3	2.6	108	93.1	105	90.5	
Mean ± SD	2.0 ± 1.3		19.7 ± 0.5		19.3 ± 1.1		F = 6593.2, *p* < 0. 001

**Table 3:** Comparison of green transformational leadership knowledge among nurse managers at pre-intervention, post-intervention, and follow-up (*N* = 116)

Green leadership knowledge	Nursing supervisors (*N* = 30)		Paired t-test 1 P1-value	Head nurses (first-line managers) (*N* = 86)		Paired t-test 2 *P*-value	Paired t-test 3 *P*-value INDEP TEST BET 2 GROUPS NOT PAIRED
	Pre	Post		Pre	Post		
	Mean ± SD	Mean ± SD		Mean ± SD	Mean ± SD		
Immediate post-intervention total Knowledge & and pre-intervention Total Knowledge	2.1 ± 0.2	19.8 ± 0.6	t1 = − 90.3, p1 < 0. 001	2.3 ± 0.5	19.7 ± 1.4	t2 = − 71.5, p2 < 0. 001	t3 = 1.6, p3 = 0.10
							t4= -1.1, p4 = 0.32
Follow-up- intervention total Knowledge & pre-intervention total Knowledge	1.1 ± 0.4	19.2 ± 1.2	t1 = − 62.8, p1 < 0.0001	2.3 ± 1.2	19.3 ± 0.9	t2 = − 65.6, p2 < 0.0001	t5 = 0.66, p5 = 0.51
Kn. Total scores: Mean ± SD	*Pre intervention* 2.1 ± 1.6			*Immediate post-intervention* 19.7 ± 0.5			Paired t test6 = − 90.3, p6 < 0. 001
Kn. Total scores Mean ± SD	*Pre intervention* 2.1 ± 1.6			*Follow up intervention* 19.3 ± 1.1			Paired t test7= − 83.3, p7 < 0. 001

**p1** = Comparison of mean knowledge score among nursing supervisors pre intervention and immediate post intervention. Also, Comparison of mean knowledge score among nursing supervisors pre intervention and follow up intervention

**p2** = Comparison of mean knowledge score among Head nurses pre intervention and immediate post intervention. Also, Comparison of mean knowledge score among Head nurses pre intervention and follow-up intervention

**p3** = Comparison of mean knowledge score pre intervention among nursing supervisors, and head nurses.

**p4** = Comparison of mean knowledge score post immediate intervention among nursing supervisors, and Head nurses.

**p5** = Comparison of mean knowledge score follow up intervention among nursing supervisors, and head nurses.

**p6 =** Comparison of mean total knowledge score among total studied nurses pre intervention and immediate post intervention.

**p7** = Comparison of mean total knowledge score among total studied nurses pre intervention and follow up intervention.

**Table 4:** Comparison of green transformational leadership practice among nurse mangers in pre intervention, post intervention, and follow up (*N* = 116)

green leadership practice	Nursing supervisors (*N* = 30)		Paired t-test 1 P1-value	Head nurses (first-line managers) (*N* = 86)		Paired t-test 2 *P*-value	*P*-value INDEP TEST BET 2 GROUPS NOT PAIRED
	Pre	Post		Pre	Post		
	Mean ± SD	Mean ± SD		Mean ± SD	Mean ± SD		
Immediate post-intervention total practice& pre-intervention total practice	14.5 ± 1.8	28.3 ± 1.02	t1 = − 53.9, p1 < 0. 001	13.1 ± 1.1	28.6 ± 0.9	t2 = − 98.3, p2 < 0.0001	t3 = 1.14, p3 = 0.25
							t4= -1.1, p4 = 0.25
Follow-up- intervention total practice & pre-intervention total practice	13.9 ± 1.2	23.2 ± 0.71	t1 = − 30.6, p1 < 0. 001	12.5 ± 1.3	23.1 ± 0.7	t2 = − 61.97, p2 < 0. 001	t5 = 1.1 p5 = 0.29
Practice total scores: Mean ± SD	*Pre intervention* 14.2 ± 1.4			*Immediate post intervention* 28.5 ± 0.9			Paired t test6 = − 13.7, p6 < 0. 001
Practice total scores: Mean ± SD	*Pre intervention* 14.2 ± 1.4			*Follow up intervention* 23.1 ± 0.7			Paired t test7= − 11.3, p7 < 0. 001

**p1** = Comparison of mean practice score among nursing supervisors pre intervention and immediate post intervention. Also, comparison of mean practice score among nursing supervisors pre intervention and follow up intervention

**p2** = Comparison of mean practice score among Head nurses pre intervention and immediate post intervention. Also, Comparison of mean practice score among Head nurses pre intervention and follow up intervention

**p3** = Comparison of mean practice score pre intervention among nursing supervisors, and head nurses (Independent t test).

**p4** = Comparison of mean practice score post immediate intervention among nursing supervisors, and head nurses (Independent t test).

**p5** = Comparison of mean practice score follow up intervention among nursing supervisors, and head nurses (Independent t test).

**p6 =** Comparison of mean total practice score among total studied nurses(*N* = 116) pre intervention and immediate post intervention. (paired t test).

**p7** = Comparison of mean total practice score among total studied nurses(*N* = 116) pre intervention and follow-up intervention. (Paired t test).

**Table 5:** Mean Score of green behavior dimensions and total green behavior, as well as green creativity among nurse managers pre-intervention, post-intervention, and follow-up (*N* = 116)

Dimensions		Pre Mean ± SD	Immediate post Mean ± SD	Follow-up Mean ± SD	F test P1	P2	P3
Green behavior	Green learning	6.3 ± 0.7	14.2 ± 0.7	11.8 ± 0.5	< 0. 001	< 0. 001	< 0. 001
	Green practice	8.3 ± 1.1	19.3 ± 0.7	15.3 ± 0.9	< 0. 001	< 0. 001	< 0. 001
	Influencing others	5.9 ± 0.8	14.3 ± 0.8	11.8 ± 0.5	< 0. 001	< 0. 001	< 0. 001
	Organizational voices	6.1 ± 1.1	14.6 ± 0.5	12.1 ± 0.3	< 0. 001	< 0. 001	< 0. 001
	Total Green behavior	26.6 ± 1.7	62.4 ± 1.4	50.9 ± 1.2	< 0. 001	< 0. 001	< 0. 001
Green creativity		11.8 ± 1.2	28.3 ± 1.1	22.3 ± 1.5	< 0. 001	< 0. 001	< 0. 001

**P1 =** comparison between the three means (pre, immediate post, and follow up) for each green behavior’ dimension, as well as Green Creativity, using Analysis of variance (F test).

**P2 =** Comparison between mean pre and Immediate post intervention for each green behavior’ dimension, total green behavior, as well as Green Creativity, using paired t test.

**P3 =** Comparison between mean pre and follow up intervention for each green behavior’ dimension, total green behavior, as well as Green Creativity, using paired t test.

**Table 6:** Level of significance and total mean score regarding pre-intervention, post-intervention, and follow-up green transformational leadership on green behavior among nurse managers (*N* = 116)

Green Transformational Leadership educational intervention	Mean difference	SD	Std. Error Mean	95% Confidence Interval of the Difference		t-test	df	Sig. (2-tailed)
				Lower	Upper			
Pre- green Transformational Leadership intervention and Immediate post -intervention on green behavior	− 35.8	2.2	0.21	− 36.2	− 35.4	− 168.5	115	^<0. 001**^
Pre- green Transformational Leadership intervention and follow up on green behavior	-24.4	2.1	0.19	-24.8	-24.0	-123.8	115	^<0. 001**^

**statistically high significant at *p* **< 0. 001**

**Table 7:** Level of significance and total mean score regarding pre-intervention, post-intervention, and follow up green transformational leadership on green creativity among nurse managers (*N* = 116)

Green Transformational Leadership educational intervention	Mean difference	SD	Std. Error Mean	95% Confidence Interval of the Difference		t-test	df	Sig. (2-tailed)
				Lower	Upper			
Pre- green Transformational Leadership intervention and Immediate post -intervention on green Creativity	− 16.5	1.8	0.17	-16.9	-16.2	-96.8	115	<0. 001**
Pre- green Transformational Leadership intervention and follow up on green Creativity	-10.5	1.9	0.18	-10.8	-10.2	-56.9	115	<0. 001**

**Statistically high significant at *p* *< 0. 001*

## Discussion

Transformational leaders play a critical role in the creation and development of a vision that inspires proactive action toward various tasks and the realization of environmental concerns and green projects. Additionally, transformational leaders assist in the development of the “creativity-enhancing forces” paradigm as well as the culture of new ideas [42]. The success and execution of creative business concepts heavily rely on the function of transformational leadership. In keeping with this idea, transformational leadership plays a critical role in fostering a sustainable environment [43]. According to [44], transformational leaders have four key behavioral traits that affect their capacity to empower followers: inspirational motivation, charismatic personality, individual attention, and cognitive stimulation.

Following the execution of the educational intervention on green transformational leadership, the current study’s findings indicated an improvement in head nurses’ overall awareness of green transformational leadership levels and their management strategies. This result is consistent with the findings of [45]. They stated that training is the most effective technique to broaden knowledge and change head nurses’ attitudes, values, and views. Additionally [46], ran a training course for first-line managers at Minia University Hospital, and they reported that it was successful in enhancing their knowledge and providing them with the support they required to carry out the management position successfully [47]. found that the educational program improved head nurses’ knowledge and emphasized the significance of their need for further training and abilities outside of what they gained in nursing school. The result was incongruent with [48] who stated that there were inadequate program changes. Furthermore, to increase program openness and replicability, planners for transformational leadership initiatives should create standard methods for designing and assessing their initiatives.

The results of this study also indicated that, compared to immediately following training, head nurses’ knowledge of green transformational leadership and related management practices slightly decreased three months later. It’s possible that some of the knowledge head nurses learned while implementing the program had been forgotten. These interpretations were corroborated by [49], who noted a deterioration in the knowledge and abilities of healthcare professionals at the National Taiwan University Hospital and emphasized the necessity for more frequent refresher training to maximize knowledge maintenance, also with [50]. In the same way [51], noted a modest reduction in nurses’ mean knowledge scores three months after program implementation compared to just after the program.

The results on green transformational leadership showed that head nurses’ employment of competitive strategies increased statistically significantly with the deployment of post-training strategies, although it remained their least favored form of green transformational leadership. This study suggested that, as a result of the program’s execution, head nurses became more conscious of and adept at using their influence. They also mastered acting with more assurance and confidence. This interpretation is supported by [52] who noted the highly statistically significant improvement and added that green transformational leadership was a crucial part of the organizational context of institutions and was concerned with putting into practice modern strategies that are attentive to environmental issues as well as increasing productivity and developing performance. Similarly [53], discovered a significant and favorable influence with regard to green transformational leadership. Moreover [54], findings showed that transformative leadership has a beneficial effect on the efficacy of leadership.

These findings have shown that head nurses should consistently implement in-service and ongoing training programs [55]. observed considerable improvement in nurse managers’ levels of utilizing competing styles post-program compared with pre-program, which lends credence to this finding. Furthermore, this result is congruent with [56] who evaluated the transformational leadership behaviors of leaders and the independent motivation of their subordinates both before and after training. The findings indicate that following the intervention, there were significant effects in the organization as measured by the subordinates’ ratings of the transformational leadership behaviors of the leaders and their self-reported autonomous motivation. This study provides theoretical insights into how transformational leadership in leaders can be enhanced or developed, which is advantageous for encouraging environmental sustainability activities inside organizations and improving employee well-being, regarding the head nurses’ overall green behavior as well as the green behavior dimensions.

According to the results of the current study, utilizing a post-training approach resulted in a statistically significant rise in the use of positive aspect strategies by head nurses. This might be attributed to the fact that the head nurses recognized the importance of green transformational leader concerns, or it could be that the training inspired head nurses to have confidence in their staff members and allow them to participate in decision-making. The findings of this study were intriguing in that they showed that, compared to a pre-training strategy, the positive aspect strategy remained high three months after deployment, but that it was slightly less than the post-training strategy. This outcome is in line with the findings of [57], who found that the program resulted in the enhancement of environmentally friendly behavior. Additionally [58], noted that leadership had a large and favorable impact on employee green behavior. The results also showed that there was no correlation between employee green behavior and environmental knowledge. This finding differs from that of [59], who discovered that workplace social context influences employees’ green behavior in a favorable but insignificant way. As a result, it is concluded that workplace social context can influence an employee’s green behavior. The results also pointed to the need for managers of the organization to make investments in raising staff understanding of environmentally friendly behavior through environmental training.

With regard to green creativity, the current study revealed a highly statistically significant rise in the usage of green transformational leadership strategies, and head nurses tended to apply them at a neutral level following the training strategy. The chief nurses continued to use the green transformational leadership approach heavily in the follow-up assessment. This could be explained by the fact that head nurses recognized the value of developing strong supervisory relationships with their nurses as a part of their duties and learned how to do so in the green transformational leadership education program. As a result, head nurses began to show greater concern and interaction with staff nurses by developing a connection with them, offering guidance and support, mentoring them to develop their abilities, and scheduling time to reflect on work and personal matters. All of this increased the head nurse’s green creativity. This finding supports the claims made by [38, 60], who argued that idealized persuasion, a commitment to environmental goals, an environmental vision, high environmental performance standards, intellectual reward, and individualized consideration can motivate staff to conduct new research and engage in environmentally friendly creative activities.

In relation to the green transformational leadership educational intervention, there was a good relationship between it and the green behavior of head nurses. The post-intervention and follow-up intervention programs showed a highly significant improvement (*p* < 0.0001) in both the individual green behavior aspects and overall green behavior. This may be a result of the leadership behaviors of those who inspire followers to perform above and beyond accepted levels of environmental performance. The findings of this study are consistent with those of [61] who investigated the connections between green transformational leadership, green human resource management techniques, and employees’ green behavior. They argued that these associations prove the beneficial connections between green transformational leadership and green behavior regarding green transformational leadership educational intervention on green creativity among head nurses. According to the current study, there was a statistically significant improvement after the educational intervention for total green transformational leadership and total green creativity among head nurses. The effectiveness of the educational intervention for green transformational leadership is favorably correlated with green creativity among head nurses. This might be explained by the study’s findings, which showed that employees’ contributions and resource acquisition led to improvements that allowed for original problem-solving. By integrating their resources, leaders can inspire their teams to work creatively and collaboratively on initiatives, which will boost their subordinates’ sense of responsibility and sense of shared purpose. This outcome is consistent with research by [62], who looked at how green leadership might inspire green innovation in university students. Students’ green creativity was greatly influenced by both green transformational leadership and green transactional leadership. Additionally, student creativity in developing green products and processes can be accomplished through the faculty members’ green leadership style. These findings concurred with [63] analysis of the function that green transformational leadership plays in encouraging green creativity through green organizational identity. They found that green transformational leadership had a beneficial impact on green organizational identity, which encouraged green creativity within the organization, by their 250 direct superiors.

Additionally [64], researched the relationship between green transformational leadership and employee green creativity and concluded that there was a positive correlation. In addition [3], asserted that green transformational leadership bolstered green innovation directly. Managers should be encouraged to improve their leadership style in order to encourage higher levels of green creativity, according to [65] study of the ongoing effects of green transformational leadership and green employee creativity among 150 employees. Additionally [66], demonstrated that the association between transformational leadership and innovative behavior of employees was positively modulated and mediated by knowledge sharing.

### Conclusion and recommendations

Knowledge, attitude, and behavior were all impacted by the educational intervention known as green transformational leadership. The program had shown a highly significant improvement (*p* < 0.0001) in both the overall and individual characteristics of green behavior. The program had a significant post-intervention impact. This study recommended that hospital directors receive training in transformational leadership to improve and enhance their managerial creativity skills in several ways:

- By developing a clear vision and mission, preserving steadfast values and high standards, having a strong sense of purpose, and having faith in both oneself and others, a leader must be able to motivate others. Additionally, they must demonstrate the importance of the human element in development and advancement by responding to individuals’ needs, maximizing their potential, and supporting them as they work to achieve their objectives.
- Leaders must explore innovative ideas and tactics in order to successfully achieve a mission. For this, it is necessary to exude charisma, set an example of desirable behavior, inspire the team, and provide support for their creativity and intellectual stimulation.
- In order to maintain consistency between their words and deeds, leaders must exhibit personal qualities including the capacity to concentrate, pay attention, adapt, and take risks. To boost morale among the team members, they must also serve as an example of how to manage workloads, communicate effectively, and reach out to others. They must also maintain a high level of harmony and collaboration within the group. Correlational and quasi-experimental study approaches are needed for future studies to investigate aspects that improve transformational leadership ability.

#### Implications for nursing management

The concept of “Green Transformational Leadership” refers to leadership behaviors and strategies aimed at promoting environmental sustainability and responsibility within an organization or a specific context. In the case we mentioned, it involves implementing educational interventions targeted at nurse managers to enhance their understanding and adoption of green practices, as well as fostering green behavior and creativity among them. In summary, the paper highlights the pivotal role of nurse managers in fostering green transformational leadership and promoting environmentally sustainable practices within healthcare settings. Nursing management should leverage the insights from this study to develop tailored strategies for educating, empowering, and supporting nurse managers in leading green initiatives and driving positive environmental change.

#### Directions for future research

Through future research, this conceptual model should be expanded upon in other fields, such as information technology. Additionally, staff nurses’ opinions of leadership style should be taken into account in future studies, as should their perspectives. Finally, the impact of a single leadership style—green transformational leadership—has been discovered by this research. Therefore, additional leadership philosophies; like servant leadership and inclusive leadership, ought to be explored in future research.

## Electronic supplementary material

Below is the link to the electronic supplementary material.

Supplementary Material 1

Supplementary Material 2

## Data Availability

The data that support the findings of this study are available from the corresponding author upon reasonable request. All data generated or analyzed during this research are included in this manuscript. The authors confirm that all methods were performed in accordance with the relevant guidelines and regulations.
