# Correction: Fostering green transformational leadership: the influence of green educational intervention on nurse managers’ green behavior and creativity

**DOI:** 10.1186/s12912-024-02423-9

**Published:** 2024-10-11

**Authors:** Manal Saleh Moustafa Saleh, Hanan Elsaid Elsabahy, Sahar Abdel-Latif Abdel-Sattar, Zaineb Naiem Abd-Elhamid, Abdulellah Al Thobaity, Sahar Mohammed Mohammed Aly, Wafaa Mohamed Shokry

**Affiliations:** 1 Nursing Administration, Faculty of Nursing, Zagazig University, Zagazig, Egypt; 2 Nursing Department, College of Applied Medical Science, Shaqra University, Shaqra, Saudi Arabia; 3 Nursing Administration, Faculty of Nursing, Mansoura University, Mansoura, Egypt; 4 Medical Surgical Nursing Department, College of Nursing, Taif University, Taif, Saudi Arabia; 5 Nursing Administration, Faculty of Nursing, Port Said University, Port Said, Egypt; 6 Nursing Administration, Faculty of Nursing, Menoufia University, Menoufia, Egypt

**Correction: BMC Nursing (2024) 23:393**

10.1186/s12912-024-01991-0

The original article presents erroneous information for affiliation #4 – the corrected affiliation information is as follows:

‘Medical Surgical Nursing Department, College of Nursing, Taif University, Taif, Saudi Arabia.’

