# Supplementary material for: Fostering green transformational leadership: the influence of green educational intervention on nurse managers’ green behavior and creativity
Source: BMC Nurs. 2024 Jun 7;23:393. doi: 10.1186/s12912-024-01991-0 (PMC11157831; doi:10.1186/s12912-024-01991-0)
Supplement: Supplementary file 1 — Supplementary Material 1 [file 12912_2024_1991_MOESM1_ESM.pdf]

The Green Transformational Leadership Knowledge Questionnaire (GTLKQ)

No	Questions	Answers		
		Correct& Complete (2)	Correct & Incomplete (1)	Incorrect (0)
1.	**Green transformational leadership is:**<br>a) Nurse managers take a series of actions to motivate subordinates to meet the requirements of environmental protection<br>b) Negative attitude toward the activities that can avoid environmental protection<br>c) None of the above<br>d) Both a & b			
2.	**Green ideas, is known as:**<br>a) The nurse manager develops an environmental vision that inspires staff nurses.<br>b) Means to pursue knowledge and practices that can lead to more environmentally friendly.<br>c) Transformational nursing managers demonstrate important concern for feelings of their nurses.<br>d) Decrease staff co-ordination and increase work load.			
3.	**All the following are Green transformational leadership elements EXCEPT:**<br>a) The nurse manager inspires the organization staff nurses with the environmental plans.<br>b) The nurse manager provides a clear environmental vision for the staff nurses to follow.<br>c) The nurse manager gets the organization members to work together for the same environmental goals.<br>d) The nurse manager Enhance performance and meet patients' needs.			
4.	**The nurse manager (leader) inspires the organization staff nurses with………………**<br>a) The patients' needs.<br>b) The environmental plans<br>c) The nursing care plan<br>d) The patients' right			
5.	**Environmental plan facilitates decision making to carry out land development with the consideration given to…………**<br>a) The Purpose, aim, goals and objectives<br>b) The natural environment, social, political, and economic factors<br>c) Performing patients' procedures on time.<br>d) Both a and c			

**6.**	**The nurse manager provides ………………. for the staff nurses to sets standards of quality:**<br>a) A clear idea<br>b) A clear objective<br>c) A clear Purpose<br>d) A clear environmental vision			
**7.**	**Environmental beliefs divided into two types are:**<br>a) Patients' needs and specific objectives<br>b) Right beliefs and wrong beliefs<br>c) General beliefs and specific beliefs<br>d) Not all of the above			
**8.**	**……………is the leadership characteristic demonstrated by a leader who acts as an environmental role model:**<br>a) Green idealized influence<br>b) Green intellectual stimulation<br>c) Green behavior<br>d) Green self-efficacy			
**9.**	**……………….. refers to a leadership characteristic denoting the ability of a nurse manager to encourage nursing staff to transcend their short-term self-interest and to strive to achieve green goals;**<br>a) Green idealized influence<br>b) Green inspirational motivation<br>c) Green intellectual stimulation<br>d) Both a and c			
**10.**	**Green behavior refers to a series of behaviors, such as;**<br>a) Protecting ecology, saving resources, and turning waste into treasure<br>b) Valuing their nursing staff' contributions to green issues<br>c) Both a and b<br>d) Not all of the above			

Cronbach's alpha for the instrument was 0.84.
