# Supplementary material for: Fostering green transformational leadership: the influence of green educational intervention on nurse managers’ green behavior and creativity
Source: BMC Nurs. 2024 Jun 7;23:393. doi: 10.1186/s12912-024-01991-0 (PMC11157831; doi:10.1186/s12912-024-01991-0)
Supplement: Supplementary file 2 — Supplementary Material 2 [file 12912_2024_1991_MOESM2_ESM.pdf]

Plan of the program

Following the development of the teaching sessions, timetables, evaluation findings, planning, and review of pertinent literature, the educational intervention was created

The general objective of the program:

This program aimed in general to raise the efficiency of green transformational leadership style through the development of information and skills of green transformational leadership style.

At the end of this program, the nurse managers will be able to:

**First session- Define the purpose of the green transformational leadership style**				
**The learning objectives at the end of the session the participant should be able to:**	**Content (Topics)**	**Teaching methods**	**Audiovisual aids:**	**Methods of evaluation**
Realize the objective of the program	-Remember expectations for the program<br>-Lists the objectives of the program	Lecture- discussion<br>Brainstorming	PowerPoint Presentation<br>Laptop.<br>Panel Paper	Feedback

**Second session- *Concept of green transformational leadership style***				
**The learning objectives at the end of the session the participant should be able to:**	**Content (Topics)**	**Teaching methods**	**Audiovisual aids:**	**Methods of evaluation**
2-1 Define of green transformational leadership style	Green transformational leadership style.	Brainstorming<br>Lecture- discussion<br>Group	Power Point Presentation<br>Panel Paper	Feedback

2-2 Identify the purpose of the green transformational leadership style	purpose of the green transformational leadership style	Group discussion	Power Point<br>Panel Paper	Feedback
2-3 Discuss the benefits of green transformational leadership style	Benefits of green transformational leadership style	Brainstorming	Power Point<br>Panel Paper	Feedback
2-4 Clarify the elements of the green transformational leadership style	Elements of green transformational leadership style	Brainstorming	Power Point<br>Panel Paper	Feedback
2-5 Identify the Overlapping meanings with green transformational leadership style	Overlapping meanings with green transformational leadership style	Lecture- discussion	Power Point<br>Panel Paper	Answer the questions
2-6 Define green transformational leadership style principles	green transformational leadership style principles	Brainstorming	Power Point<br>Panel Paper	Answer the questions
2-7 Discuss Functions of green transformational leadership style	Functions of green transformational leadership style	Brainstorming	Power Point<br>Panel Paper	Feedback

**Third session-** ***elements of green transformational leadership and its relation green behavior and green creativity***				
**The learning objectives at the end of the session the participant should be able to:**	**Content (Topics)**	**Teaching methods**	**Audiovisual aids:**	**Methods of evaluation**
2-8 List elements of green transformational leadership and its relation green behavior and green creativity	elements of green transformational leadership.	Demonstrating videos	Power Point<br>Presentation<br>Panel Paper	Feedback
2-9Discuss the factor influencing green transformational leadership style	factor influencing green transformational leadership style	Demonstrating videos	Power Point<br>Panel Paper	Feedback
2-10 Discuss enabling Factors For Effective green	Eenabling factors for effective green	Demonstrating videos	Power Point<br>Panel Paper	Feedback

transformational leadership style	transformational leadership style			
2-11 List Barriers To Sustaining green transformational leadership style	Barriers To Sustaining green transformational leadership style	Brainstorming Lecture- discussion	Power Point Panel Paper	Answer the questions

**Fourth session-** **green transformational dimensions,**				
**The learning objectives at the end of the session the participant should be able to:**	**Content (Topics)**	**Teaching methods**	**Audiovisual aids:**	**Methods of evaluation**
3-1 Define the green transformational leadership style	Concept of the green transformational leadership style	Brainstorming discussion	Power Point Presentation Panel Paper	Feedback
3-2 List criteria for an effective green transformational leadership style	Criteria for an Effective green transformational leadership style	Lecture- discussion	Power Point Panel Paper	Feedback
3-3 Identify and characteristics of green transformational leadership style	Characteristics green transformational leadership style	Lecture- discussion	Power Point Panel Paper	Feedback
3-4 Identify important characteristics of green transformational leadership style	Important characteristics of green transformational leadership style	Brainstorming Lecture- discussion	Power Point Panel Paper	Feedback
3-5 Discuss green transformational dimensions,	green transformational dimensions,	show scenario-based situations on four aspects of dimensions.	Power Point	Answer the questions
3-6 Defined important guidance for green transformational leadership style	Important guidance for green transformational leadership style	Lecture- discussion	Power Point Panel Paper r	Answer the questions

**Fifth session- successful green transformational leadership styles .**				
**The learning objectives at the end of the session the participant should be able to:**	**Content (Topics)**	**Teaching methods**	**Audiovisual aids:**	**Methods of evaluation**
Discuss successful green transformational leadership styles	- Communication- Coaching- Problem solving and decision making legation- Motivation-	Demonstrate role-playing	Power Point Presentation Laptop.	Answer the questions

	Conflict resolution- Stress management- Time management			

**Fifth session- successful green transformational leadership styles (Communication- Coaching-)**				
**The learning objectives at the end of the session the participant should be able to:**	**Content (Topics)**	**Teaching methods**	**Audiovisual aids:**	**Methods of evaluation**
4-1Define the communication	Concept of Communication	Lecture-	Power Point Presentation Panel Paper	Feedback
4-2 Explain communication process	Process of the communication	Brainstorming discussion	Power Point Panel Paper	Feedback
4-3 Identify barriers for communication	Barriers of the communication.	Brainstorming	Power Point Panel Paper	Feedback
4-5 List tips for being active listener	Becoming an active listener	Brainstorming	Power Point Panel Paper	Feedback
4-6 Define the Coaching	Definition of the coaching	Lecture- discussion	Power Point Panel Paper	Feedback
3-6 List ten tips for Successfully Coaching Employees.	Ten keys for successfully coaching employees.	Brainstorming Lecture- discussion	Power Point Panel Paper	Answer the questions

**Fifth session- successful green transformational leadership styles (delegation - motivation - problem solving- decision making)**				
**The learning objectives at the end of the session the participant should be able to:**	**Content (Topics)**	**Teaching methods**	**Audiovisual aids:**	**Methods of evaluation**
4-7 Define the delegation	Definition of the delegation.	Brainstorming	Power Point<br>Panel Paper	Feedback
4-8 Identify benefits of delegation.	Benefits of delegation	discussion	Power Point<br>Panel Paper	Feedback
4-9 Discuss delegation process	Delegation process	Brainstorming<br>Lecture-discussion	Power Point<br>Panel Paper	Feedback
4-10 Define the motivation	Definition of the motivation.	Brainstorming<br>Lecture-discussion	Power Point<br>Panel Paper	Feedback
4-11 Discuss how supervisor can motivate employee	How supervision can motivate?	Small group	Pens +papers	Practical applications
4-12 Define problem solving	Definition of the problem solving.	Brainstorming	Power Point	Practical applications
4-13 Define decision making	Definition of the decision making.	Brainstorming	Power Point<br>Panel Paper	Answer the questions
4-14List factors influencing decision making processes	- Factors influencing decision making process	Brainstorming	Power Point<br>Panel Paper	Answer the questions
4-15Clarify the steps of problem solving /Decision Making process	Problem solving/Decision making process.	Small group	Pens +papers	Practical applications

**Fifth session- successful green transformational leadership styles (conflict resolution-stress management)**				
**The learning objectives at the end of the session the participant should be able to:**	**Content (Topics)**	**Teaching methods**	**Audiovisual aids:**	**Methods of evaluation**
4-16 Define conflict	Definition of the conflict	Lecture	Power Point Panel Paper	Feedback
4-17 List Causes of Conflict	Causes of conflict	Brainstormin g	Power Point Panel Paper	Answer the questions
4-18 Identify signs of workplace Conflict	Conflict Signs of workplace	Brainstormin g	Power Point Panel Paper	Answer the questions
4-19 Clarify the ways of addressing conflict	Steps for conflict resolution.	Small group	Pens +papers	Practical applications
4-20 Discuss steps for Conflict Resolution	Ways of addressing conflict/ Conflict Styles/ Strategies	Brainstormin g Lecture-discussion	Power Point Panel Paper	Answer the questions
4-21 Define stress	Definition of stress.	Brainstormin g	Power Point Panel Paper	Answer the questions
4-22 List causes of stress	Causes of stress.	Brainstormin g	Power Point Panel Paper	Answer the questions
4-23 Identify Possible signs of stress	Signs of excess stress	Brainstormin g	Power Point Panel Paper	Answer the questions
4-24 Discuss how supervisors can minimize organizational' stressors	How supervisor can minimize organization, stressor ?	Brainstormin g	Power Point Panel Paper	Answer the questions
4-25 Define time management	- Definition of time management.	Brainstormin g	Power Point Panel Paper	Answer the questions
4-26 Identify advantages of time management	Advantages of time management	Brainstormin g	Power Point Panel Paper	Feedback

4-27 Discuss techniques and Skills for time management:	Techniques and skills for time management	Brainstorming discussion	Power Point Panel Paper	Feedback
4-28 Define time wasters	Time wasters.	Brainstorming	Power Point Panel Paper	Feedback
4-29 Discuss possible solutions of time wasters	Possible solutions of time wasters	Brainstorming	Power Point Panel Paper	Feedback
4-30 Identify top time tips.	Top time tips	Brainstorming	Power Point Panel Paper	Feedback

**Sixth session- green ideas and carry out brainstorming to solve environmental problem (Implementing green transformational leadership style).**				
**The learning objectives at the end of the session the participant should be able to:**	**Content (Topics)**	**Teaching methods**	**Audiovisual aids:**	**Methods of evaluation**
5-1 Clarify the standard of green transformational leadership style	Standards of green transformational leadership style	Lecture	Power Point Panel Paper	Feedback
5-2 Explain the process of green transformational leadership style	**Process of green transformational leadership style:**<br>A - Introductory stage.<br>B - Implantation stage.<br>C – Consolidation stage.	Brainstorming	Power Point Panel Paper	Feedback

5-3-Explain the six stages of green transformational leadership style	**Stage of green transformational leadership style**<br>1-Problem identification.<br>2-Objective practice analysis.<br>3- Setting objective.<br>4- Planning.<br>5- Implementation.<br>6- Evaluation.	Small group	Pens +papers	Practical applications
5-4 Discuss Proctor's Models of green transformational leadership style	**Models of green transformational leadership style:**	Lecture-discussion	Power Point<br>Panel Paper	Feedback

**5- Implementing green transformational leadership style**				
**The learning objectives at the end of the session the participant should be able to:**	**Content (Topics)**	**Teaching methods**	**Audiovisual aids:**	**Methods of evaluation**
5-5 Discuss Derby's Models of green transformational leadership style	**Models of green transformational leadership style**	Lecture	Power Point<br>Panel Paper	Feedback
5-6Discuss Holistic Nursing Model of green transformational leadership style	**Models of clinical supervision:**	Small group	Pens +papers<br>Power Point<br>Panel Paper	Practical applications
5-7 Discuss rights and responsibilities in supervisor-green transformational leadership style	Rights and responsibilities of the supervisor and	Brainstorming	Power Point<br>Panel Paper	Feedback

	supervisee in green transformational leadership style			
5-8 Discuss Ethical, Legal issues and Confidentiality in green transformational leadership style	Ethical, legal issues and confidentiality in green transformational leadership style.	Brainstorming Lecture-discussion	Power Point Panel Paper	Feedback
5-9 Clarify the importance of documenting and contract in green transformational leadership style	Documentation of green transformational leadership style.	Lecture-discussion	Power Point Panel Paper	Feedback
